# How low can they go when going with the flow? Tolerance of egg and larval fishes to rapid decompression

**DOI:** 10.1242/bio.017491

**Published:** 2016-05-26

**Authors:** Craig A. Boys, Wayne Robinson, Brett Miller, Brett Pflugrath, Lee J. Baumgartner, Anna Navarro, Richard Brown, Zhiqun Deng

**Affiliations:** 1 New South Wales Department of Primary Industries, Port Stephens Fisheries Institute, Taylors Beach Road, Taylors Beach, New South Wales 2316, Australia; 2 Institute of Land, Water and Society, Charles Sturt University, Elizabeth Mitchell Drive Thurgoona, New South Wales 2640, Australia; 3 New South Wales Department of Primary Industries, Narrandera Fisheries Centre, 64 Buckingbong Road Narrandera, New South Wales2700, Australia; 4 Water Research Laboratory, University of New South Wales, 110 King Street Manly Vale, New South Wales2093, Australia; 5 Pacific Northwest National Laboratory, Richland, WA 99354, USA

**Keywords:** Barotrauma, Hydropower, Larval drift, Murray-Darling basin, Physoclistous, Piecewise regression

## Abstract

Egg and larval fish that drift downstream are likely to encounter river infrastructure and consequently rapid decompression, which may result in significant injury. Pressure-related injury (or barotrauma) has been shown in juvenile fishes when pressure falls sufficiently below that at which the fish has acclimated. There is a presumption that eggs and larvae may be at least as, if not more, susceptible to barotrauma injury because they are far less-developed and more fragile than juveniles, but studies to date report inconsistent results and none have considered the relationship between pressure change and barotrauma over a sufficiently broad range of pressure changes to enable tolerances to be properly determined. To address this, we exposed eggs and larvae of three physoclistic species to rapid decompression in a barometric chamber over a broad range of discrete pressure changes. Eggs, but not larvae, were unaffected by all levels of decompression tested. At exposure pressures below ∼40 kPa, or ∼40% of surface pressure, swim bladder deflation occurred in all species and internal haemorrhage was observed in one species. None of these injuries killed the fish within 24 h, but subsequent mortality cannot be excluded. Consequently, if larval drift is expected where river infrastructure is present, adopting design or operational features which maintain exposure pressures at 40% or more of the pressure to which drifting larvae are acclimated may afford greater protection for resident fishes.

## Abstract (precis)

**Summary:** Eggs are extremely tolerant to rapid decompression, although larvae may be injured once pressure falls below 40% of the pressure to which they are acclimated.

## INTRODUCTION

The downstream drift of eggs and larvae is a long-distance dispersal strategy common to many fish species (Brown and Armstrong, 1985; Humphries et al., 2002; Penaz et al., 1992). In many river systems such a dispersal strategy is likely to expose large numbers of eggs and larvae to hazardous conditions as they encounter river infrastructure, such as weirs and hydropower turbines. Large numbers of eggs and larvae (often close to 100%) of different species can be killed when passing weirs (Baumgartner et al., 2006; Marttin and De Graaf, 2002) or hydropower turbines (many examples reviewed in Marcy et al., 1978). It is likely that such extreme mortality rates can reduce recruitment of individuals to adult populations, particularly in rivers where river infrastructure is prevalent and the impact may be cumulative.

To ensure that eggs and larvae have the best chance of recruiting to and sustaining adult populations, river infrastructure needs to be designed and operated with fish welfare in mind, but this cannot be done if the factors responsible for their injury and mortality are not properly understood. Barotrauma resulting from rapid decompression has long been suspected to be a major cause of injury and mortality at river infrastructure (e.g. Marcy et al., 1978). Rapid decompression can occur when fish pass weirs because they move instantaneously from depth under a sluice gate up to the water surface (with surface pressure being ∼101 kPa), with slight sub-atmospheric pressures (∼95 kPa) likely under the gate (Boys et al., 2013). Even larger degrees of decompression are possible at other infrastructure, for example, pressures as low as 7 kPa have been reported at Kaplan turbines, although average values of ∼24 kPa are more typical (Deng et al., 2010).

Barotrauma in fish can result from rapid decompression and can be caused by rapidly expanding undissolved gas in the swim bladder and other organs, or when the solubility of gas in blood decreases, causing gas to be released into tissues (Feathers and Knable, 1983). Reports of barotrauma injury in juvenile fish are common and include swim bladder rupture, eye dislocation (exophthalmia), and haemorrhaging or bubble formation (emphysema) in vasculature, brain, gills, or heart (Beyer et al., 1976; Brown et al., 2012b; Cramer and Oligher, 1964; Pflugrath et al., 2012). Susceptibility of juvenile fish to barotrauma can vary among species and in recent years studies have modelled injury or mortality of different species over a broad range of pressure changes, which has allowed criteria to be developed to identify relatively safe ranges of pressure changes (Boys et al., 2016; Brown et al., 2012b).

There is reason to believe that eggs and larvae may also be susceptible to barotrauma injury, but this is far from resolved. Eggs have been shown to be vulnerable to damage when exposed to rapid pressure increases (Chourrout, 1984; Goudie et al., 1995), but not after rapid pressure drops (Beck et al., 1975; Brown et al., 2013). As for larvae, many species inflate their swim bladders at very early stages, meaning that they are likely to contain undissolved gas at the time of downstream larval drift (Battaglene and Talbot, 1990; Rowland, 1996). This, combined with the fragile or poorly developed nature of internal organs and vasculature of larval fish, has led to the proposition that larvae may be more susceptible to barotrauma injury than older juvenile fish (Brown et al., 2014).

Published literature examining the susceptibility of larval fishes to barotrauma is inconsistent. Larval mortality has been shown to be high in some species but low in others when exposed to sub-atmospheric pressure (Cada et al., 1982; Ginn et al., 1978; Kedl and Coutant, 1976). There are a number of problems that arise when interpreting these studies. Firstly, they all used an apparatus that did not allow the impacts of rapid decompression to be isolated from those of shear stress. Secondly, they did not subject fish to pressures below 50 kPa (half surface pressure); meaning they cannot account for the more severe ranges of decompression that may be experienced at some hydropower turbines. Finally, in these studies it was not possible to accurately define the pressure that a fish was exposed to, but instead a broad range of possible pressures may have been experienced. As such, mortality rates cannot be associated with specific levels of decompression, and therefore it is impossible to determine when benign levels of decompression become more damaging.

To address these limitations, we used a barometric chamber to expose eggs and larvae of three physoclistous species to rapid decompression over a broad range of discrete pressure changes. This paper will show that although eggs are unaffected by barotrauma, there is evidence that swim bladder deflation and internal haemorrhage can occur in some larvae. As we will discuss, this finding has important implications for how weirs and hydropower turbines should be designed and operated in areas of known larval drift.

## RESULTS

**Fig. 1.**
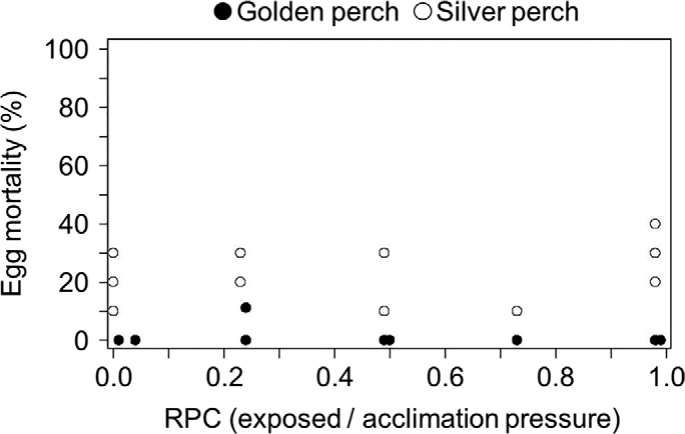
**The probability that silver perch and golden perch eggs will die before hatching after being exposed to simulated infrastructure passage across a range of ratio of pressure changes (RPC_E/A_).** Each point represents the percentage of that test group (10 eggs) affected.

Hatching success was unaffected by ratio of pressure change (RPC) for both silver perch and golden perch eggs (golden perch χ^2^=0.001, d.f.=1, *P*=0.993; silver perch χ^2^=0.677, d.f.=1, *P*=0.41; Fig. 1).

**Fig. 2.**
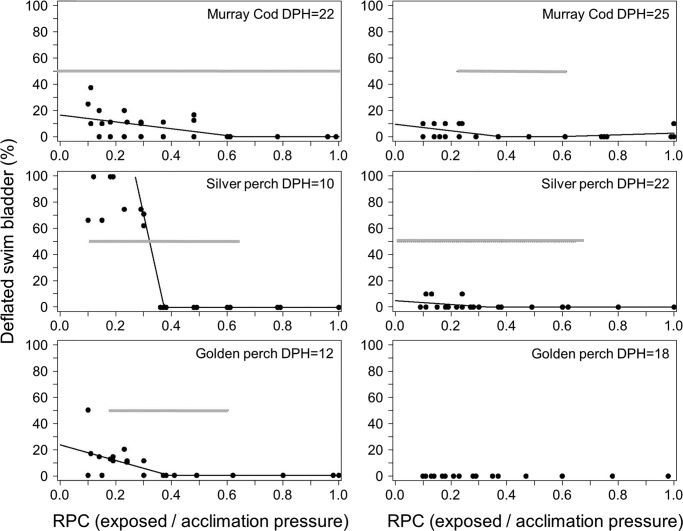
**The percentage of larval Murray cod (top), silver perch (middle) and golden perch (bottom) with a deflated swim bladder at two different ages (days post hatch, DPH) following simulated infrastructure passage over a range of ratio of pressure changes (RPC).** Piecewise regression lines are shown if there was convergence in the piecewise linear regression model and the relationship were statistically significant. The grey line shows the band between the 95% confidence intervals of the breakpoint outlined in Table 1.

Three types of injury were observed in larval fish: swim bladder deflation, internal emphysema and internal haemorrhaging. Deflation of the swim bladder contributed a large amount of the total injury response in larvae (Fig. 2). For 10-day-old silver perch, 12-day-old golden perch and 25-day-old Murray cod swim bladder deflation began below a RPC threshold of ∼0.4. Deflation may occur at slightly higher RPC (∼0.6) in 22-day-old Murray cod, but large confidence intervals indicate a large degree of uncertainty surrounding this estimate (Fig. 2, Table 1). The magnitude of increase in swim bladder deflation was substantially higher for 10-day-old silver perch than all other larvae studied (Fig. 2).

**Table 1.**
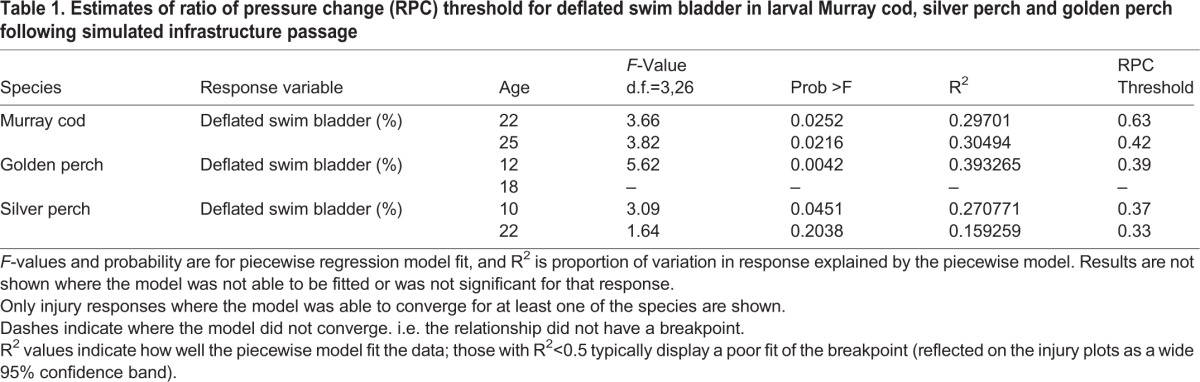
**Estimates of ratio of pressure change (RPC) threshold for deflated swim bladder in larval Murray cod, silver perch and golden perch following simulated infrastructure passage**

**Fig. 3.**
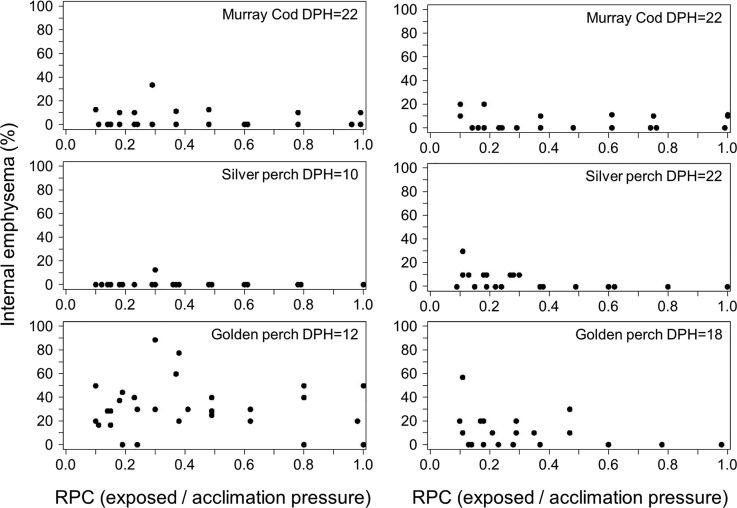
**The percentage of larval Murray cod (top), silver perch (middle) and golden perch (bottom) with internal emphysema at two different ages (days post hatch, DPH) following simulated infrastructure passage over a range of ratio of pressure change (RPC).** Piecewise regression lines are not shown because there was no convergence of models.

**Fig. 4.**
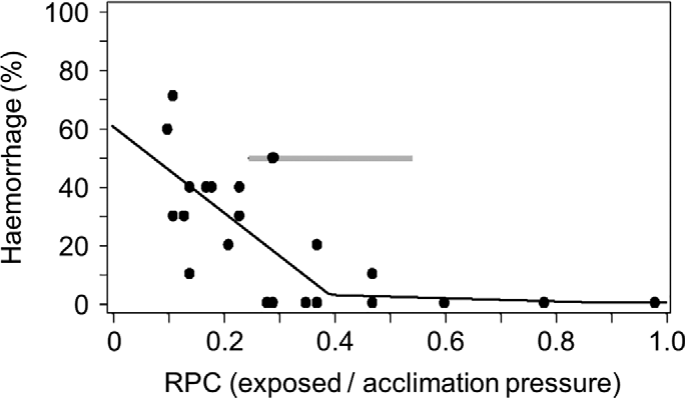
**The percentage of 18 DPH golden perch with internal haemorrhaging following simulated infrastructure passage over a range of ratio of pressure change (RPC).** The grey line shows the band between the 95% confidence intervals of the breakpoint estimated using piecewise regression.

Internal emphysema (see Fig. S1) were observed in many fish including handling controls, however this is not likely to be related to rapid decompression because no significant relationship could be identified for this response and RPC (Fig. 3). In 18-day-old golden perch, internal haemorrhaging was observed with blood pooling in the cavity posterior to the swim bladder (see Fig. S1). Haemorrhaging increased significantly as RPC fell below an estimated threshold of 0.39 (piecewise regression, *F*=16.5, d.f.=3, 26, *P*<0.0001; R^2^=0.66; Fig. 4).

**Fig. 5.**
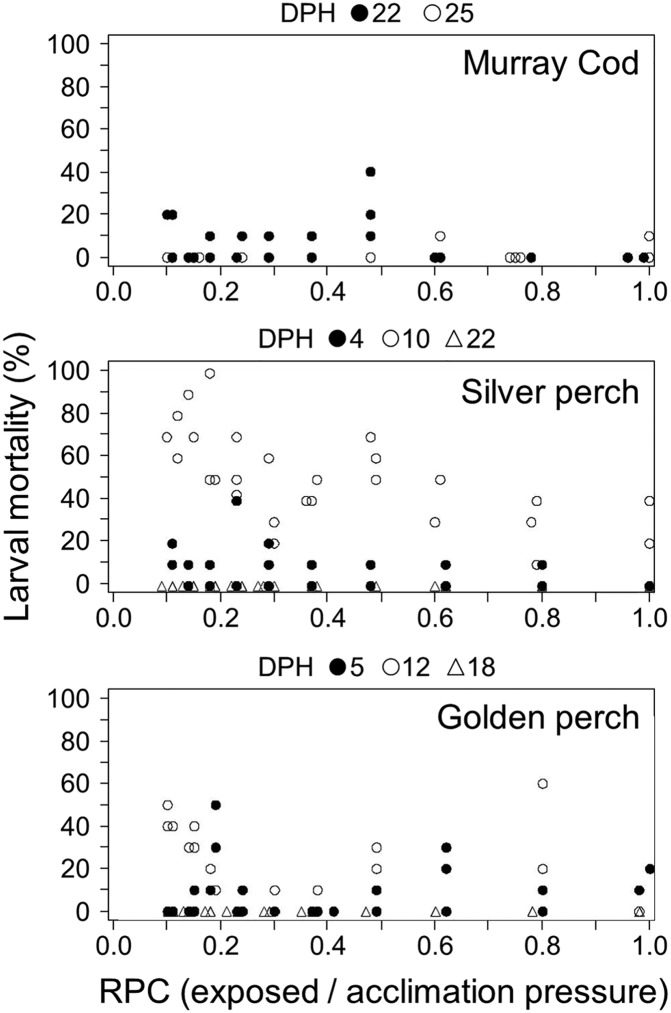
**The percentage of larval Murray cod, silver perch and golden perch dead within 24 h of simulated infrastructure passage at different ages (days post hatch, DPH) and across a range of ratio of pressure changes (RPC_E/A_).** Each point represents the percentage of that test group (10 larvae) affected.

Despite these observed injuries, we found little evidence that the level of decompression was associated with an increase in short-term (within 24 h) mortality of larvae. For all three species, age (but not RPC) was significantly associated with larval mortality (Table 2). In other words, the severity of decompression did not impact larval mortality although some ages were more susceptible to death than others through experimental handling. The highest mortality was found in 12-day-old golden perch and 10-day-old silver perch (Fig. 5). For 5-, 12- and 18-day-old golden perch, the average rates of mortality were 7.7, 16.3 and 0%, respectively. Murray cod mortality averaged 5% (22-days-old) and 1.7% for (25-days-old), while silver perch had average mortalities of 7, 50 and 0% for 4-, 10- and 22-day-olds, respectively.

**Table 2.**
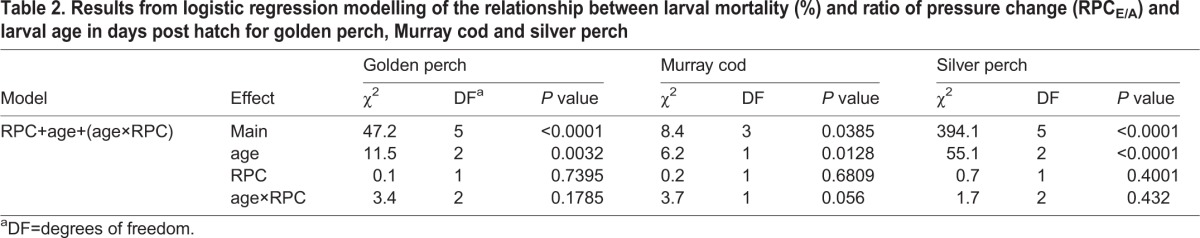
**Results from logistic regression modelling of the relationship between larval mortality (%) and ratio of pressure change (RPC_E/A_) and larval age in days post hatch for golden perch, Murray cod and silver perch**

## DISCUSSION

Based on the evidence in this study, we present three propositions relating to the role of barotrauma in injuring and killing eggs and larval fish at river infrastructure: (1) eggs are not susceptible to barotrauma; (2) larvae are susceptible to injury only once pressure falls below ∼40% of the pressure to which they are acclimated; (3) none of these injuries appear to kill larvae within 24 h, but subsequent mortality cannot be excluded.

### Eggs are not susceptible to barotrauma

It appears beyond doubt that eggs can experience rapid decompression and still successfully hatch. We found no evidence that the hatch rate of golden perch or silver perch was impaired at any of the levels of decompression that were tested. Moreover, little mortality has been reported in previous studies where eggs have been exposed to similarly low pressures [e.g. striped bass (RPC=0.14); Beck et al., 1975], or substantially lower pressures [e.g. white sturgeon (RPC=0.05); Brown et al., 2013]. The tolerance of eggs to rapid decompression is almost certainly related to the fact that they do not contain free gas that could expand when pressure is reduced. Instead pelagic eggs contain fluids which are minimally compressible compared to gas and their buoyancy is typically attained through the presence of lipid globules (Craik and Harvey, 1987), as is the case with golden perch (Anderson et al., 1990).

### Larvae are susceptible to barotrauma injury

A novel feature of our study was that we exposed larvae over a sufficiently broad range of discrete pressure drops to provide evidence that larval Murray cod, golden perch and silver perch are susceptible to injury only once pressure drops below a RPC∼0.4, that is ∼40% of the pressure to which they are acclimated. This is a smaller pressure drop than is required to cause swim bladder rupture in juvenile Murray cod (∼0.2 RPC is required), but a larger pressure drop than is required to cause swim bladder rupture in juvenile silver perch (0.8 RPC is required) (Boys et al., 2016 ). However, the injury threshold of 0.4 RPC identified for larvae by our research is relatively similar to thresholds reported previously for Chinook salmon from the USA. For example, juvenile salmon exposed to simulated turbine passage began to show damage such as swim bladder rupture and haemorrhaging when exposed to pressures 40-50% of acclimation pressure, which resulted in swim bladder gas expansion of between 2.5-2.2 times (Stephenson et al., 2010). In another study it was noticed that injuries and mortality increased sharply once the swim bladder doubled and tripled in size (Brown et al., 2012a).

Given that no other studies have examined a sufficiently broad range of pressure change in other larval fishes, we cannot make direct comparison between the threshold provided here and that of larvae of other species because such thresholds have yet to be developed. That being said, evidence from other studies do not necessarily contradict our proposition of a 0.4 RPC threshold for larvae. The larvae of common carp (*Cyprinus carpio*) (Ginn et al., 1978), bluegill (*Lepomis macrochirus*), largemouth bass (*Micropterus salmoides*) and channel catfish (*Ictalurus punctatus*) (Cada et al., 1981; Kedl and Coutant, 1976) have shown little or no mortality when decompressed to less severe levels than the proposed threshold (∼50 kPa or RPC∼0.5). Moreover, 9-day-old white sturgeon (*Acipenser transmontanus*) (Brown et al., 2013) were found to have a greater propensity for gut herniation and death if exposed to pressures as low as 5 kPa (RPC=0.05), which is a substantially more extreme level of decompression than the injury threshold we present.

The white sturgeon example (Brown et al., 2013) illustrates an important feature of larval susceptibility to barotrauma. That is that susceptibility can vary with age and some larvae may only be susceptible within a certain ‘window’ or stage. Silver perch and golden perch were substantially more likely to be injured when less developed (10- and 12-days-old, respectively) than when more fully developed (22- and 18-days-old). Although it has been previously shown that larvae can be more susceptible to physical stresses at smaller sizes (Cada et al., 1982), ours is the first study to demonstrate an inverse relationship between larval age and barotrauma in isolation of other physical stresses, and illustrates the importance of considering the age structure of the larval drift when determining the potential risk of barotrauma at river infrastructure.

Swim bladder deflation and internal haemorrhage were two injuries associated with rapid decompression, although neither led to mortality of larvae within 24 h of exposure, possibly because the injuries were not life threatening or because fish were not observed for long enough to account for delayed mortality. In another study, largemouth bass, smallmouth bass and common carp larvae were observed to have progressively higher mortality beyond 24 h, when observed for up to 72 h, after passing through a pump and condenser tube (Cada et al., 1982).

There is reason to believe that swim bladder deflation may indeed result in delayed mortality. Swim bladder deflation beyond RPC∼0.4 suggests that expanding the swim bladder by 2.5 times exceeds the upper volumetric limit in these species. Over-expansion ruptures the swim bladder of juvenile Murray cod and silver perch (Boys et al., 2016), as well as many other species (Brown et al., 2012b; Jones, 1951). However, there is reason to believe that, in our study, deflation did not result from rupture. Swim bladder rupture is typically associated with the presence of internal emphysema (Brown et al., 2012b) because bubbles of gas released from the swim bladder become trapped in the viscera or embedded in surrounding tissue. Although we observed some cases of internal emphysema in larvae, it was seldom associated with the occurrence of a deflated swim bladder and, in some instances, control fish (that were not decompressed) also had emphysema.

An alternative explanation is that the swim bladder deflated when gas was vented through the mouth. Such a phenomenon has been referred to as ‘expelling’ or ‘burping’ gas from the swim bladder and is typically reported in physostomic fish because they possess a pneumatic duct linking the swim bladder to the gut (Brown et al., 2012b). ‘Burping’ may make physostomous species less susceptible to barotrauma injury than physoclistous species because it can release gas from the swim bladder without rupture; however, to date, there has not been enough different fish species or sizes examined to determine that this is the case. Murray cod, silver perch and golden perch are all physoclistic species and have a ‘closed’ swim bladder without a pneumatic duct as juveniles and adults; however we were not able to find evidence in the literature, or through our own observations, to exclude the possibility that a rudimentary duct may still exist in young larvae. The presence of such a rudimentary duct in early-stage larvae has been observed in other physoclistous species (Bulak and Heidinger, 1980), allowing for initial swim bladder inflation by gulping air at the surface (Battaglene et al., 1994; Battaglene and Talbot, 1990; Chapman et al., 1988; Hadley et al., 1987).

Regardless of whether the larvae of physoclistic species are capable of ‘burping’ when decompressed, this may not necessarily translate into greater survival at a hydropower turbine. The reason for this is that physoclistic species that fail to initially fill their swim bladder at early-larval stages rarely regain this ability and can suffer reduced growth, skeletal deformities and increased susceptibility to stress-induced mortality (Al-Abdul-Elah et al., 1983; Spectorova and Doroshev, 1976; Weppe and Bonami, 1983). Therefore, it is possible that early-stage physoclistic larvae that are forced to burp during decompression may never regain the capacity to refill the swim bladder, and as a result suffer longer-term effects on development, growth or survival. This hypothesis needs to be tested by holding larvae for longer than 24 h after decompression, preferably to a point after juvenile metamorphosis.

### Implications for the design of river infrastructure

Accepting the proposition that larvae are minimally injured if not subjected to pressures less than 40% of that to which they are acclimated (RPC of 0.4), recommendations can be made as to the likely cause of injury at weirs and hydropower turbines, and how best to mitigate them. As previously mentioned, undershot weirs are unlikely to generate pressures lower than 95 kPa (Boys et al., 2013), therefore a surface drifting larvae would not experience a pressure less than 94% of that to which it was acclimated (95 kPa÷101 kPa=RPC of 0.94), that is, not exceeding what appears to be the injury threshold. Moreover, a larvae would need to be acclimated and drifting at a depth in excess of 14 m (238 kPa) to be exposed to a sufficiently large pressure drop to exceed the threshold for injury (95 kPa÷238 kPa=an RPC of 0.4). Such a depth of drift is very unlikely to be encountered in the Murray-Darling basin, where these three species come from because there are few reaches in the Murray-Darling basin (outside of reservoirs) that exceed this depth. This may not be the case for all river systems in the world, such as the Mekong river, which has extensive larval drift and extensive deep pools (Conlan, 2014). Again, this illustrates that if one wishes to apply the findings of this study to other river systems or species, it is important to understand how the migratory behaviour of the species in question may differ.

If decompression at weirs within the Murray-Darling basin is unlikely to be sufficient to cause barotrauma, previous reports of significant larval mortality of Murray cod (>50% mortality), silver perch (>90%) and golden perch (>90%) after passing an ‘undershot’ weir structure (Baumgartner et al., 2013, 2006) are therefore unlikely to related to pressure. Instead they may be due to other hydraulic stresses such as fluid shear stress, which can occur in turbulent flow immediately downstream of weirs (Boys et al., 2013). Laboratory studies have revealed that egg and larvae of the three species studied here are extremely vulnerable to damage when exposed to even moderate increases in shear stress (Boys et al., 2014).

When compared to weirs, there is much more likelihood that hydropower turbines will exceed the 0.4 RPC threshold noted here and there are many of these throughout most river systems in the world. Much lower pressures of ∼24 kPa are typical of Kaplan turbines (Deng et al., 2010), which would be sufficient to far exceed the proposed injury threshold for even surface drifting larvae, corresponding to a RPC of 0.2. The extent of decompression that fish are exposed to through turbines can vary depending on turbine design and operations such as the discharge, head, and efficiency (Deng et al., 2010). Research is needed to better understand the pressure conditions created by a greater range of turbine designs and operational scenarios than has been undertaken to date.

### Conclusion

Fish eggs can withstand levels of decompression far in excess of what they are likely to encounter if entrained at river infrastructure; however, larvae of the Australian physoclistous species studied here are generally susceptible to injury beyond a RPC of ∼0.4, or when exposed to pressures less than 40% of that to which they are acclimated whilst drifting. Additionally, it cannot be discounted that some of these injuries may prove fatal in the medium term. Based on this evidence, and in the absence of injury thresholds for the larvae of other species, it would be prudent for those who design or operate river infrastructure to ensure decompression does not exceed this tolerable limit in areas of known larval drift. Exceeding this limit is unlikely at weirs, but is substantially more likely at hydropower turbines. If pressures are expected to exceed tolerable limits at a hydropower facility in an area of known larval drift, it may be appropriate to minimise larval entrainment by either using screening devices at intakes or by limiting their operation during fish spawning periods.

## MATERIALS AND METHODS

### Species studied

Three species of fish from the Murray-Darling basin (Australia) were studied: golden perch (*Macquaria ambigua* Richardson, 1845), Murray cod (*Maccullochella peelii* Mitchell, 1838), and silver perch (*Bidyanus bidyanus* Mitchell, 1838), all which have larvae that exhibit extensive downstream drift (Humphries and King, 2004; Humphries et al., 2002; Koehn and Harrington, 2005). Additionally, golden perch and silver perch have buoyant eggs which drift downstream (Reynolds, 1983; Tonkin et al., 2007). The eggs of Murray cod were not investigated since they are demersal and do not drift downstream, but instead are under parental care in a nest.

All three species are recognised as vulnerable or threatened under State or National legislation, with river regulation recognised as a significant key threatening process. Murray cod and silver perch have particularly declined in association with a rapid increase in river regulation (Gehrke et al., 1995), and there is evidence that their larvae are highly susceptible to mortality during downstream passage through weirs (Baumgartner et al., 2013, 2006). Golden perch are still widespread throughout the Murray-Darling basin although they have declined in some areas where weirs and dams have created barriers to migration; like the other two species, larvae can die in significant numbers during downstream passage through weirs (Baumgartner et al., 2013).

All procedures performed on animals were in accordance with the ethical standards approved by the Fisheries NSW Animal Care and Ethics Committee, authority number ACEC 12/05.

### Fish handling

#### Eggs

Adult golden and silver perch were induced to spawn at the Narrandera Fisheries Centre during their natural reproductive season (January to February 2013) using Ovaprim^®^(Aquatic Diagnostic Services International Ptd Ltd, Banora NSW Australia), an analogue of salmon gonadotropin-releasing hormone. Adults spawned two days after induction and the eggs were collected and placed in an aerated 15 litre bucket. Within 24 h, viable eggs were siphoned from their bucket using vinyl tubing and held in aerated 700-ml plastic jars. Ten eggs were placed in each jar, each jar corresponding to a test group of fish (further explanation of these test groups follows). Chamber experiments subsequently began within 24 h.

#### Larvae

Surplus silver perch and golden perch eggs were hatched for the larval experiments. Experiments were carried out on 4-, 10- and 22-day-old (post hatch) silver perch larvae and 5-, 12- and 18-day old golden perch larvae. Once hatched, silver perch larvae were kept prior to experimentation in aerated trays (50 cm long, 50 cm wide and 15 cm deep). From three days old, the larvae were fed newly hatched *Artemia nauplii* and a pulverised commercial pellet. Unlike silver perch larvae, golden perch are more difficult to maintain in hatchery conditions, and therefore at three days old golden perch were stocked into a large earthen pond (3600 m^2^ and ∼3 ml) and allowed to feed naturally on plankton. Twenty-four hours prior to experimentation, larvae were collected from the pond with a hand net and placed in aerated trays (as above). They were kept in the trays until needed for the pressure chamber experiments. During this time, no supplementary food source was provided.

Murray cod larvae were experimented at 22 and 25 days old, after the time at which they would be expected to have left the nest in the wild. Eggs were harvested from spawning boxes in earthen ponds in November 2012 and kept for 7–10 days in incubating tanks until hatched. Larvae were then moved into aerated trays (as above) until needed for the pressure chamber experiments. The larvae were fed daily on newly hatched *A. nauplii*, but only from 17 days of age, because prior to that age they still retain stores of yolk (King, 2002).

Approximately 12–24 h before experimentation, the larvae of all three species were individually siphoned using vinyl tubing from their holding trays into aerated 700-ml plastic jars. Ten larvae were placed in each jar, with each jar corresponding to a different test group of fish.

All tanks or trays holding parental fish, eggs, or larvae were supplied with flow-through, bore-drawn water (∼10 l min^−1^). Daily water quality measurements were taken from the larvae holding trays, supply tank and barometric chamber (see below). Throughout the study, the mean (±s.e.m.) pH was 8.08±0.02 (range of 7.95–8.24); conductivity was 14.6±1.38 ms cm^−1^ (9.40–20.10); dissolved oxygen was 7.15±0.28 mg l^−1^ (5.07–7.67); total dissolved gas saturation was 101.41±0.34% (99.72–101.86); and temperature was 18.46±0.23°C (17.0–19.10).

### Decompression in barometric chamber

The eggs and larvae of silver perch and golden perch, but only the larvae of Murray cod, were rapidly decompressed in a barometric chamber (see Boys et al., 2016 for a detailed description of chamber design and operation). Prior to testing, the larvae were acclimated to surface pressure (where ∼101 kPa indicates surface pressure and 0 kPa indicates vapour pressure). Fish were rapidly decompressed from this pressure, to one of ten discrete nadir (lowest) pressures. By doing this, decompression was examined over a range of discrete ratios of pressure change (RPC; i.e. exposure pressure÷acclimation pressure; *sensu* Boys et al., 2016) (Table 3). Decompression was rapid (an example pressure profile is shown in Fig. S2), occurring at speeds between 0.1 and 0.5 s or equating to a rate of pressure change of between 63.9 and 359.1 kPa s^−1^. Whilst this is an extremely rapid rate of decompression, rates in excess of this can be experienced by fish at river infrastructure such as undershot weirs (Boys et al., 2013) and hydropower facilities (Deng et al., 2010).

**Table 3.**
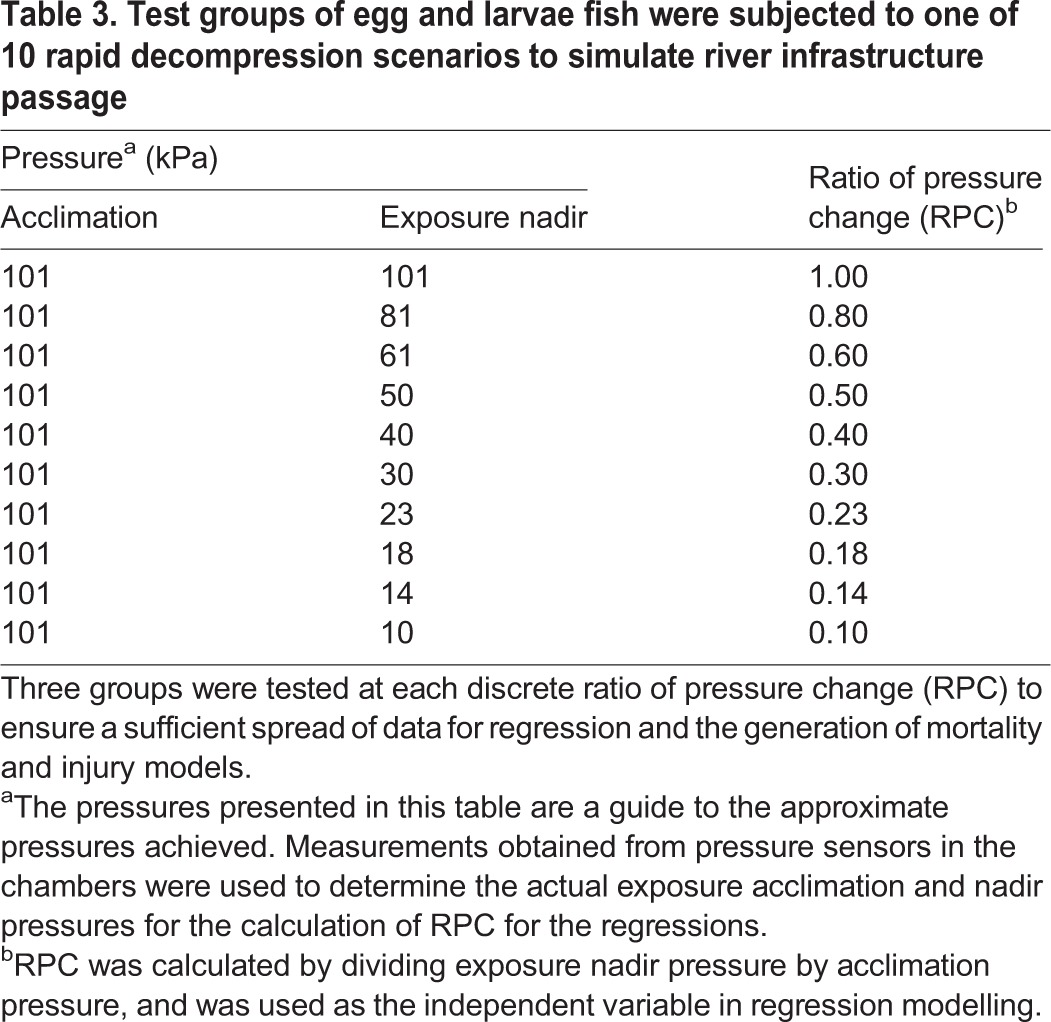
**Test groups of egg and larvae fish were subjected to one of 10 rapid decompression scenarios to simulate river infrastructure passage**

#### Eggs

A single jar of 10 eggs (corresponding to a test group) was placed in the chamber for decompression. This involved replacing the plastic lid of a jar with a fine mesh cover and sealing the jar in the chamber for 5 min at atmospheric pressure (∼101 kPa), before subsequently decompressing to one of 10 possible nadir pressures, ranging from 101 kPa (no pressure change, serving as a control) to as low as ∼10 kPa (Table 3). Following a return to atmospheric pressure, each jar of eggs was removed from the chamber and placed back into the holding trays, under aeration, for 48 h. After this time, the eggs were examined to determine how many had successfully hatched and how many had died.

#### Larvae

Silver perch, golden perch and Murray cod larvae were rapidly decompressed in a barometric chamber as per the experimental design outlined for the egg experiments (Table 3). Before decompression, a single test group of fish was gently poured from its holding jar into a clear acrylic cylinder (11 cm diameter and 20 cm high) with a mesh screen lid and placed into the chamber. The cylinder ensured that larvae could be easily observed and retrieved from the chamber after the experiment. Following decompression, larvae were returned to their individual aerated jars. The number of dead larvae in each test group was counted immediately following decompression and then again at 24 h post experimentation. At the 24-h point, any larvae still alive were euthanised using a solution of 100 mg l^−1^ ethyl-p-amino benzoate (benzocaine), transferred to a Petri dish, and examined under a dissecting microscope fitted with a digital camera to determine the presence of external and internal injuries. It was very difficult and usually impossible to ascertain injuries in the youngest age class of larvae (i.e. 4-day-old silver perch and 5-day-old golden perch), and for these ages only mortality was assessed and compared to control groups. In the older age classes, the injuries looked for included exophthalmia (eye dislocation); decapitation; internal haemorrhaging; gut herniation or expulsion of digestive contents; emphysema (gas bubbles) in the body cavity, eyes or fins; and evidence of swim bladder rupture. As the larvae were too small to identify rupture points in the swim bladder, a visible swim bladder was assumed to be intact and inflated, whereas the absence of a visible swim bladder was assumed to be deflated and thereby an indication of rupture.

### Statistical analyses

The percentage of eggs or larvae injured or dead within a test group of 10 fish was treated as the dependent variable. For the egg experiments, logistic regression was used to determine whether the total mortality rate was influenced by RPC. For the larval experiments, logistic regression models were first fitted including age (that is, days post hatch) as a fixed factor. For larval injury, data were also analysed using linear piecewise regression (Toms and Lesperance, 2003 as applied by Boys et al., 2016). The piecewise approach generated ‘broken-stick’ models, where two lines of different slope join at a ‘breakpoint’. The breakpoint was used as an objective means of estimating thresholds in RPC where a significant increase in the probability of injury was first detected. Such an approach has proven successful in determining thresholds for barotrauma injury in juveniles Murray cod and silver perch (Boys et al., 2016).
